# The type III secretion system translocases IpaC, SipC, and BipC are partially folded alpha helical proteins lacking in tertiary structures

**DOI:** 10.1371/journal.pone.0331455

**Published:** 2025-09-12

**Authors:** Jacob M. Kroh, Amritangshu Chakravarty, Supratim Dey, Roberto N. De Guzman

**Affiliations:** Department of Molecular Biosciences, University of Kansas, Lawrence, Kansas United States of America; Islamic Azad University, IRAN, ISLAMIC REPUBLIC OF

## Abstract

The Type III secretion system (T3SS) is essential in the virulence of many bacterial pathogens that cause infectious diseases in humans. The T3SS consists of an injectisome that bacteria use to inject virulence proteins directly into human cells to initiate infection. Part of the injectisome is the translocon, which forms a pore on the host membrane to allow the passage of virulence proteins into the host. The translocon is assembled from two membrane proteins, termed major and minor translocases, based on their relative sizes from each other. Both major and minor translocases are essential for virulence. The atomic structure for any of the minor translocases remains unknown. Prior results from circular dichroism (CD), structural modeling, and AlphaFold predictions suggested these proteins have three-dimensional structures as alpha helical bundles. We have expressed and purified the T3SS translocases IpaC, SipC, and BipC from *Shigella*, *Salmonella*, and *Burkholderia*, respectively. Our results of CD spectroscopy, thermal denaturation, and 2D NMR show that IpaC, SipC, and BipC are alpha helical proteins, but they lack tertiary structures. The highest level of protein structures for these translocases are secondary structures. IpaC and SipC are predominantly alpha helical, whereas BipC also contains a significant amount of random coil conformation. Our results suggest that the translocon is assembled from proteins that lack tertiary structures.

## Introduction

Many pathogens assemble the type III secretion system (T3SS) to inject virulence proteins into human cells to cause infectious diseases [1–3]. The T3SS consists of a nanoscale injectisome that is essential in the pathogenesis many Gram-negative bacteria that contribute to millions of infections worldwide [4,5]. The injectisome is assembled from over 20 different proteins and it consists of a base, a needle, a tip, and a translocon. The translocon is formed by two membrane proteins – the minor and major translocases where ‘minor’ and ‘major’ refer to their relative sizes. IpaC [6–13], SipC [6,14,15], and BipC [16–19] are the minor translocases of *Shigella*, *Salmonella*, and *Burkholderia*, respectively; and they are predicted to contain 1 transmembrane helix [6]. Advances in the past two decades from cryo-EM, crystallography, and NMR have defined in atomic detail nearly every part of the injectisome [2], however, the atomic structure of the translocon remains unknown. The atomic structures for any of the T3SS minor translocases are currently unknown. Hence, even basic membrane protein structural data for the translocases remains unclear. Part of the problem is difficulty in expression and purification of the minor translocases and their tendency to aggregate in solution.

IpaC has been expressed and purified by others in 3 expression vectors – with a His-tag in pET-15b [13]*,* as a fusion protein with thioredoxin in pET-32b [13]*,* and by co-expression with its chaperone IpgC in pACYCDuet-1 [8]*.* IpaC in pET-15b contains 20 extra residues at the N-terminus consisting of a His-tag and a thrombin cleavage site [13]. IpaC in pET-15b is insoluble when induced at 37 ºC, 2 h, but forms insoluble and soluble fractions in approximately equal amounts when induced at 30 ºC, 2 h [13]. Insoluble IpaC from pET-15b was purified by nickel affinity chromatography under denaturing conditions using 6 M urea followed by dialysis in urea-free buffer [9–11,13,20], whereas soluble IpaC was purified by nickel affinity chromatography under native condition [12]. IpaC in pET-32b consists of thioredoxin (Trx, 109 residues) as a solubility enhancer, a His-tag, and a thrombin cleavage site at the N-terminus of IpaC. The Trx-IpaC fusion protein was soluble when expressed at 37 ºC, 3 h [13]. IpaC was co-expressed with its chaperone IpgC, using two plasmids – His-tag IpgC in pET-15b and IpaC in pACYCDuet-1 [8]. The IpaC-IpgC complex was soluble and was purified under native conditions by nickel affinity chromatography. To dissociate IpaC from IpgC, the pH was dropped to pH 5, followed by gel filtration to separate IpaC (363 residues) from IpgC (155 residues). The pH was then raised to pH 7 to obtain soluble IpaC [8]. IpaC co-expressed in pACYCDuet-1 [8] was also purified under urea denaturing conditions, followed by detergent solubilization of IpaC [7]. These constructs allowed the characterization by CD of IpaC purified under non-denaturing conditions [8] and IpaC stabilized in Tween 20 [20] or in 200 mM urea [7,9].

SipC has been expressed and purified similar to IpaC in pET-15b with a 20-residue N-terminal sequence containing a His-tag and a thrombin site [11]. Recombinant SipC was insoluble and required purification in 6 M urea, followed by dialysis in urea-free buffer. Purified SipC aggregated and was kept soluble in 2 M urea and rapidly diluted in urea-free buffer just prior for characterization by fluorescence spectroscopy [11]. Recombinant BipC was expressed in pET-30a [17], which appended a 48-residue tag at the N-terminus consisting of a His-tag, a thrombin site, and an enterokinase site. Recombinant BipC was soluble and was purified by nickel affinity chromatography followed by characterization by CD and thermal denaturation [17].

For biophysical studies, it is desirable to express recombinant proteins with minimal cloning artifact and purified without chaotropic reagents such as urea. The challenges of protein expression and purification of these translocases have hampered biophysical characterization of these proteins. Here, we report the expression of IpaC, SipC, and BipC. The constructs we used contained shorter cloning artefact and tags. Our purification protocols resulted in the complete removal of urea and allowed the acquisition of CD and 2D ^1^H-^15^N NMR data. Our CD and NMR results revealed that these minor translocases are alpha-helical proteins, however, they lack three-dimensional structures.

## Materials and methods

### Expression and purification of IpaC

Full-length *Shigella flexneri* (strain SHIFL) IpaC was subcloned in the NdeI/XhoI sites of pET-22b which appended an 8-residue (LEH_6_) His-tag at the C-terminus to generate the expression plasmid pAC1. For protein expression, pAC1 was transformed into *E. coli* BL21(DE3). A single colony was used to inoculate a 20 mL LB starter culture with carbenicillin (100 µg/mL) and grown overnight at 37 °C with shaking. The starter culture was used to inoculate 1 L TB containing carbenicillin (100 µg/mL) and grown at 37 °C with shaking. At OD_600_ ~ 0.6, protein expression was induced with 1 mM isopropyl-β-D-thiogalactopyranoside (IPTG), and cell growth was continued at 37 °C for 4 hours. OD_600_ at harvest was ~ 1.6–1.9. Cells were harvested by centrifugation at 2824 × g (4,000 rpm in a Beckman JA10.5 rotor) at 4 °C for 20 minutes. Cell pellets were resuspended at 4 °C in binding buffer (20 mM Tris-HCl pH 8.0, 500 mM NaCl, 5 mM imidazole) with 1 mM phenylmethylsulfonyl fluoride (PMSF) as broad-spectrum serine protease inhibitor. Cells were lysed by sonication at 4 °C using a Fisher Model 500 Sonic Dismembrator fitted with 0.5 inch flat tip operating at 20 kHz, with a total time duration of 30 min at 30% amplitude in pulses of 2 sec on, 2 sec off power. The cell lysate was centrifuged at 20442 × *g* (13,000 rpm in a Beckman 25.5 rotor), 4 °C for 20 minutes. Full length IpaC went into inclusion body. This process was repeated twice – the supernatant was discarded, and the cell pellet was resuspended in 20 mL binding buffer with 1 M urea and 1% Triton X-100, and pelleted by centrifugation at 20442 × *g*, 4 °C for 20 min. The inclusion body pellet was resuspended in 30 mL binding buffer with 6 M urea but without Triton X-100, incubated at room temperature in a platform rocker for 30 min, and centrifuged at 20442 × *g*, 4 °C for 30 min. The supernatant was loaded in a 5 mL nickel column and washed 3 times with 30 mL of binding buffer with 6 M urea. IpaC was eluted from the column with a total of 45 mL of buffer (20 mM Tris-HCl pH 8.0, 500 mM NaCl, 250 mM imidazole, 6 M urea). Fractions containing purified IpaC were pooled and dialyzed in 1L buffer without urea (20 mM Tris-HCl pH 8.0, 500 mM NaCl), and dialyzed twice in 1L buffer (20 mM potassium phosphate pH 7.4, 50 mM NaCl). Urea was further removed using Amicon Ultra-15 3K centrifugal filters at 4 °C, 3220 × *g* (4000 rpm on an Eppendorf A-4–62 rotor), 15 minutes, a total of 6 times with a 7^th^ time to concentrate the protein. Protein concentration was determined by absorbance at A_280_.

### Expression and purification of IpaC NTD

The N-terminal domain (NTD) of IpaC (residues 1–100) was subcloned in the NdeI/XhoI sites of pET-22b to generate the expression plasmid pAC2. IpaC NTD expressed mainly as inclusion bodies and was expressed and purified following the protocol described above for full-length IpaC. IpaC NTD in pAC2 can be expressed either at 25 °C overnight in BL21(DE3) Codon Plus or at 37 °C for 4 hr in BL21(DE3). IpaC NTD expressed in BL21(DE3) at 37 °C for 4 hr yield a mix estimated of about 15% soluble fraction and 85% inclusion bodies. Similar to the protocol for full-length IpaC above, the IpaC NTD inclusion bodies were solubilized in 6 M urea for purification by nickel affinity chromatography followed by exhaustive dialysis in urea-free buffer.

### Expression and purification of IpaC CTD

The C-terminal domain (CTD) of IpaC residues 173−363 was subcloned as a fusion protein in the plasmid pDZ1 [21] to generate the expression plasmid pAC3. The fusion protein expressed by pAC3 consisted of a His-tag, the GB1 domain for solubility, a Tobacco Etch Virus (TEV) protease cleavage site (ENLYFQS), and IpaC residues 173−363. For protein expression, pAC3 was transformed into *E. coli* BL21(DE3), and a single colony was used to inoculate a 20 mL LB starter culture and grown at 37 °C overnight. The starter culture was used to inoculate a 1 L TB culture media containing carbenicillin (100 µg/mL), and cells were grown at 37 °C with shaking. At OD_600_ ~ 0.6, protein expression was induced with 1 mM IPTG and cell growth was continued at 37 °C for 4 hr. Cells were harvested by centrifugation at 2824 × g at 4 °C for 20 minutes (typical OD_600_ at harvest was ~ 1.6–1.9). Cell pellets were resuspended at 4 °C in binding buffer with 1 mM PMSF protease inhibitor and sonicated as above. The cell lysate was centrifuged at 20442 × *g*, 4 °C for 20 minutes. To the supernatant was added 700 µL of 5% polyethyleneimine (PEI) to precipitate the nucleic acids and nucleic acid binding proteins, followed by centrifugation at 20442 × *g*, 4 °C for 20 min. The supernatant was loaded onto a 5 mL Ni^2+^ affinity column and washed 3× with 30 mL of buffer (20 mM Tris-HCl pH 8.0, 500 mM NaCl, 10 mM imidazole). The fusion protein His_6_-GB1-IpaC^173−363^ was eluted from the nickel column with 45 mL of buffer (20 mM Tris-HCl pH 8.0, 500 mM NaCl, 250 mM imidazole). Fractions containing purified fusion protein were pooled with 250 µL of 0.04 mM recombinant TEV protease, transferred to a dialysis bag (MWCO 3000), and placed in 1 L buffer (20 mM Tris-HCl pH 8.0, 20 mM NaCl, 0.5 mM EDTA, 1 mM DTT) at room temperature overnight. This step cleaved the His_6_-GB1 tag and released IpaC^173−363^ from the fusion protein. The dialysis bag was transferred into 1 L buffer (20 mM Tris-HCl pH 8.0, 500 mM NaCl, 5 mM imidazole) to remove DTT and EDTA from the digest. The digest was then passed through a 5 mL Ni^2+^ affinity column and washed 3× with 30 mL of buffer (20 mM Tris-HCl pH 8.0, 500 mM NaCl, 10 mM imidazole). The His_6_-GB1 tag bound to the Ni^2+^ column while IpaC^173−363^ passed through and was collected and dialyzed into buffer (20 mM potassium phosphate pH 7.4, 50 mM NaCl). Protein was concentrated using Amicon Ultra-15 3K and protein concentration was estimated by A_280_.

### Expression and purification of SipC

Full-length *Salmonella typhimurium* SipC was subcloned into the NdeI/XhoI sites of a linearized pET-22b vector by In-Fusion Cloning (Takara Bio) to generate the plasmid pJK14. This construct appended an 8-residue (LEH_6_) His-tag at the C-terminus of SipC. SipC was expressed and purified following a similar protocol above used for IpaC in pAC1. SipC was expressed in a 2L culture with 1 mM IPTG at 37 °C, 4 hr. Similar to IpaC, recombinant SipC formed inclusion bodies, and was solubilized in 6 M urea for purification by nickel affinity chromatography and dialyzed two times in urea-free buffer. Upon removal of urea, a white, fluffy precipitant formed in the dialysis bag. The precipitant was separated from the supernatant by centrifugation at 4 °C, 3220 × *g* for 30 minutes and the supernatant was removed without disturbing the pellet. Residual urea was further removed and the protein was concentrated using Amicon Ultra-15 3K centrifugal filters at 4 °C, 3220 × *g*, 15 minutes, 7 times, as described above for full-length IpaC. Protein concentration was determined by absorbance at A_280_.

### Expression and purification of BipC

DNA encoding BipC, BipC NTD residues 1−200, or BipC CTD residues 197−419 was PCR amplified from *Burkholderia pseudomallei* genomic DNA (strain K96243) and subcloned into the NdeI and BamHI sites of the expression vector pDZ1 [21], to generate the plasmids pSD1, pSD2, and pSD3, respectively. pDZ1 was derived from pET-21a and appends a His_6_-tag, a GB1 domain as a solubility enhancement tag, and a tobacco etch virus protease cleavage site at the N-terminus of target protein. After digestion by TEV protease, the target protein contains 2 N-terminal residues, GH, as cloning artefacts. For protein expression, plasmids were transformed into *E. coli* BL21-CodonPlus (DE3)-RIPL (Agilent), and grown overnight in 10 mL LB. The overnight growth was centrifuged (3220 × *g*, 15 min) and the cell pellet was resuspended into 1L M9 minimal media supplemented with 100 μg/mL carbenicillin and 24 μg/mL chloramphenicol. Cells were grown in a 37^o^C shaker and induced with 1 mM IPTG at A_600_ ~ 0.6–0.8 and cell growth was continued overnight in a 15^o^C shaker to a final A_600_ ~ 2.0. Cells were harvested by centrifugation (2,700 × *g*, 10 min), resuspended in 30 mL binding buffer (500 mM NaCl, 20 mM Tris-HCl, 5 mM imidazole, pH 8.0), and sonicated in the presence of 300 μL of 1 mM PMSF. The cellular debris was pelleted by centrifugation (18,200 × *g*, 12 min), and 600 μL of 5% polyethyleneimine was added to the supernatant to precipitate the nucleic acids followed by another centrifugation (18,200 × *g*, 12 min). The supernatant was loaded onto a 5 mL Ni^2+^-affinity column that was pre-equilibrated with binding buffer, the column was washed with five column volume of wash buffer (500 mM NaCl, 20 mM Tris-HCl, 15 mM imidazole, pH 8.0) and protein was eluted with elution buffer (500 mM NaCl, 20 mM Tris-HCl, 300 mM imidazole, pH 8.0). Fractions containing purified BipC were combined and dialyzed overnight in 1 L of TEV buffer (20 mM NaCl, 50 mM Tris-HCl, 5 mM EDTA, 1 mM DTT, pH 8.0) in the presence of 250 μL of 0.06 mM recombinant TEV protease to digest the fusion protein. The digest was dialyzed again in binding buffer and purified by a second round of Ni^2+^-affinity chromatography as described above. Purified BipC eluted in the wash fractions, whereas the His_6_-tagged GB1 domain was retained in the column. BipC NTD and BipC CTD were also expressed and purified similarly as above. Purified proteins were dialyzed in buffer (20 mM NaH_2_PO_4_/ Na_2_HPO_4_, pH 6.8, 100 mM NaCl) and concentrated using Amicon Ultra 3K (Millipore). Protein concentrations were estimated by using the Bradford assay.

### Expression and purification of IpaB NTD

The IpaB N-terminal domain (NTD), residues 74–224, with wild-type or N142C mutation, was expressed and purified as previously described [22]. The cysteine-point mutation did not alter the secondary structure of IpaB NTD [22].

### Circular Dichroism spectroscopy

Far-UV Circular Dichroism (CD) spectroscopy was acquired on a Jasco J-185 CD spectrometer using a 1 mm pathlength quartz cuvette. Protein samples for far-UV CD were typically diluted to a final concentration of 0.1–0.2 mg/mL, in 5–10 mM potassium phosphate buffer pH 7.4, and 5–10 mM NaCl. CD spectra were acquired as the average of five scans and recorded in triplicate at 25 °C, from a wavelength range of 190–260 or 195–260 nm at a scan rate of 50 nm/min, maintaining a HT voltage less than 600 V to improve signal to noise. Thermal denaturation was performed by monitoring the molar ellipticity at 222 nm from 25 °C and 80 °C with a temperature ramp rate of 5 °C/min. CD spectra were plotted in R. Near-UV CD spectroscopy was acquired under similar conditions. Protein samples were diluted to a final concentration of 0.28–0.56 mg/mL, in 15 mM potassium phosphate buffer at pH 7.4, and 15 mM NaCl. Data were acquired from a wavelength range of 260–310 nm in a 1 cm pathlength. Urea-denaturation of IpaC and IpaB NTD were performed as described above on a wavelength range of 210–260 nm with freshly prepared 10 M urea that was diluted to final concentration and incubated at room temperature before each scan.

### NMR spectroscopy

Uniformly ^15^N-labeled proteins for 2D NMR were obtained following the protocols above for protein expression and purification, except, the culture media used was M9 minimal media supplemented with 1g/L ^15^NH_4_Cl as the sole source of nitrogen. Protein samples for NMR were typically 0.1 to 0.3 mM protein concentration, in buffer (20 mM NaH_2_PO_4_/ Na_2_HPO_4_, pH 6.8, 100 mM NaCl) and 10% (v/v) D_2_O. Two dimensional ^1^H-^15^N TROSY NMR spectra [23] were acquired using a Bruker Avance 800 MHz spectrometer equipped with a cryogenic triple resonance probe at 20 °C. Data were processed with NMRPipe [24] and analyzed using NMRView [25].

## Results

### Expression and purification of full-length IpaC

**Fig 1:**
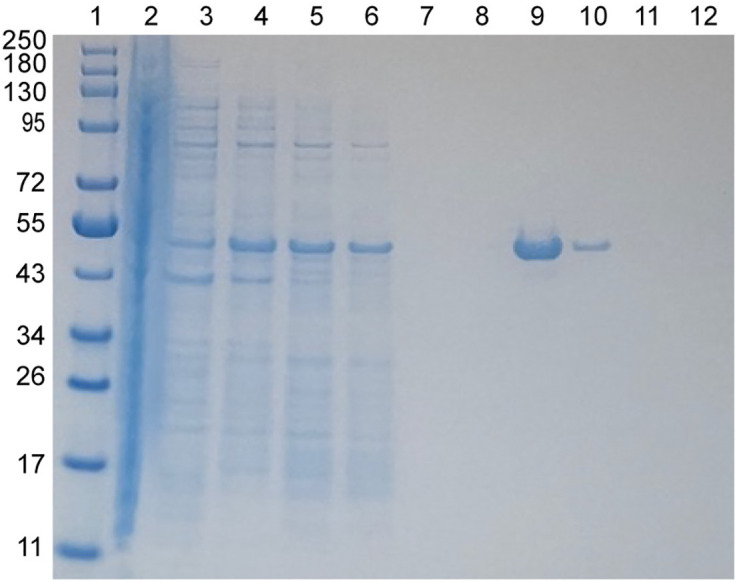
Purification of IpaC by nickel affinity chromatography. Lanes: 1 molecular weight marker, 2-4 supernatants of pellets washes, 5 flowthrough, 6-8 washes, 9-11 elutions, 12 column strip with 0.5 M EDTA.

Full-length IpaC in pET-22b appended a short, non-cleavable 8-residue His-tag at the C-terminus of IpaC to facilitate one-step purification by nickel affinity chromatography. This construct represents the shortest tag among the various expression constructs of IpaC in the literature [8,13]. IpaC was expressed as inclusion bodies and required solubilization in 6 M urea for a one-step purification by nickel affinity chromatography (Fig 1). Urea was removed from the solution by stepwise dialysis into buffers without urea. It is worth noting that a white, fluffy precipitate formed in the dialysis bag upon the removal of urea in this manner. We estimated this change took place around 200 mM urea which is consistent with previously reported findings [7,9]. However, there is a fraction of IpaC as verified by SDS-PAGE that remained in solution upon decanting, typically around 0.5–1 mg/mL from a 1 L culture. This fraction can be buffer exchanged using Amicon centrifugal filters as many times as we wished with no further precipitation observed (Fig 1).

### Expression and purification of IpaC NTD and IpaC CTD

**Fig 2:**
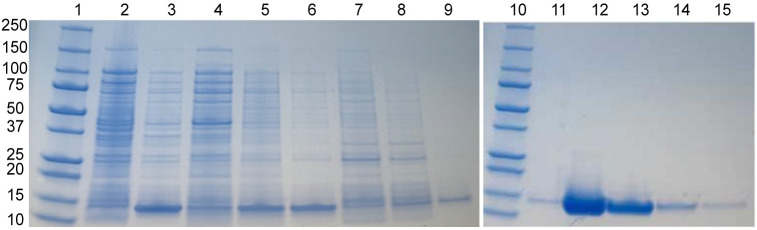
Purification of IpaC NTD (residues 1-100) by nickel affinity chromatography. Lanes: 1 molecular weight marker, 2 before IPTG induction, 3 after IPTG induction, 4-6 supernatants of pellets washes, 7 flowthrough, 8-9 washes, 10 molecular weight marker, 11 wash, 12-14 elutions, 15 column strip with 0.5 M EDTA.

**Fig 3:**
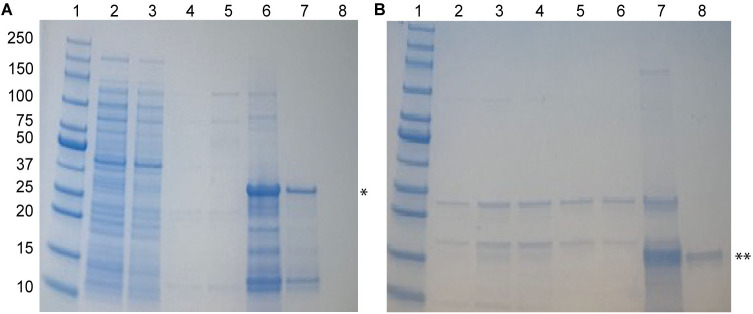
Purification of IpaC CTD (residues 173-363). (A) First purification of IpaC CTD by nickel affinity chromatography. Lanes: 1 molecular weight marker, 2 supernatant, 3 flowthrough, 4, 5. washes, 6-8. Elution of His-GB1-TEV- IpaC CTD *. (B) Second purification of IpaC CTD by nickel affinity chromatography after TEV protease digestion. Lanes: 1 marker, 2 supernatant, 3 flowthrough, 4-6 washes, 7-8 elution IpaC CTD**.

We expressed and purified the N-terminal domain, IpaC NTD (residues 1–100); and the C-terminal domain, IpaC CTD (residues 173–373). Unlike IpaC, IpaC NTD did not go 100% into inclusion bodies when expressed at 37 °C 4 hr in *E. coli* BL21(DE3), but the ratio was estimated to be 85:15 inclusion bodies to soluble fraction. IpaC NTD was somewhat more soluble than the full-length IpaC, yielding typically 3–4 mg/mL protein from a 1 L culture (Fig 2). On the other hand, IpaC CTD expressed as a fusion protein with GB1 yielded a soluble protein that allowed purification under native conditions (Fig 3). Upon TEV protease digestion, IpaC CTD remained soluble in solution.

### Expression and purification of SipC

**Fig 4:**
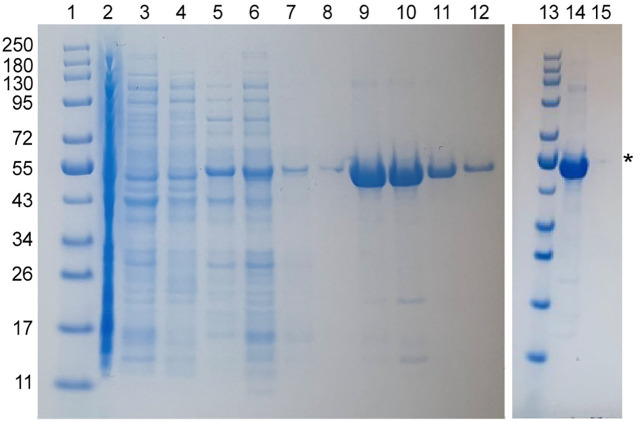
Purification and solubility of SipC. SDS-PAGE of SipC inclusion bodies solubilized in 6 M urea and purified by nickel affinity chromatography. Lanes: 1 molecular weight marker, 2-4 supernatants of pellets washes, 5 flowthrough, 6-8 washes, 9-11 elutions, 12 column strip with 0.5 M EDTA, 13 molecular weight marker, 14 insoluble SipD, 15 soluble SipC*. The fractions from 9-11 were pooled and stepwise dialyzed to remove urea. SipC precipitated upon removal of urea. After separation of precipitant from the supernatant by centrifugation, the precipitate was resuspended in buffer containing 6 M urea and analyzed by SDS-PAGE. The supernatant showed a faint protein band indicating a soluble fraction of SipC.

Following similar protocol described above for IpaC, we expressed and purified SipC in pET-22b, which appended a non-cleavable 8-residue His-tag with the sequence LEH_6_ at the C-terminus of SipC. This construct is the shortest possible tag on SipC available in the literature [11] and should be useful for experimental approaches that require recombinant SipC with the least number of cloning artefacts. Compared to IpaC, SipC is much less soluble in urea-free buffers, forming a white precipitate upon dialysis in urea-free buffers. Nevertheless, the supernatant contained a small amount of soluble SipC (Fig 4) estimated at about 0.1 mg/mL (2 uM) from a 2 L culture by A_280_, which enabled the acquisition of CD data for SipC.

### Expression and purification of BipC, BipC NTD, and BipC CTD

**Fig 5:**
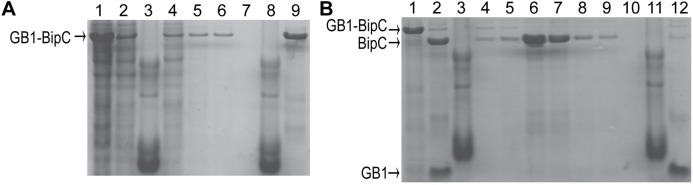
Purification of BipC from the GB1-BipC fusion protein. (A) Purification of the fusion protein GB1-BipC by nickel affinity chromatography. Lanes: 1 supernatant, 2 flowthrough, 3 molecular weight marker, 4-6 washes, 8 marker, 9 elution of GB1-BipC. (B) Purification of BipC by nickel affinity chromatography after TEV protease digestion of the fusion protein GB1-BipC. Lanes: 1 fraction before TEV protease digestion, 2 fraction after TEV protease digestion, 3 molecular weight marker, 4-9 washes containing BipC, 11 molecular weight marker, 12 elution of His-tagged GB1 domain.

Among the three T3SS minor translocases described herein, BipC is unique in that it is soluble, and it can be expressed and purified under native conditions. To obtain BipC under native conditions, BipC was expressed as a fusion protein with the GB1 domain as a solubility tag. The GB1 domain has a His-tag for purification by nickel affinity chromatography, and TEV protease cleavage site to allow recovery of recombinant BipC following digestion with recombinant TEV protease (Fig 5). The recombinant BipC acquired using this method had a 2-residue cloning artifact with the sequence GH. Using the GB1 fusion protein also allowed us to express and purify the BipC NTD and CTD fragments under native conditions, and both domains were soluble. BipC can be expressed using pET-22 in *E. coli* BL21(DE3) at 37 °C 4 hr, however it formed inclusion bodies under these conditions. Following solubilization of the BipC inclusion bodies in 6 M urea and purification by nickel affinity chromatography, there was proteolytic cleavage that resulted in smaller fragments of BipC. BipC in pET-22b is thus not an ideal expression system for BipC.

### CD of IpaC

**Fig 6:**
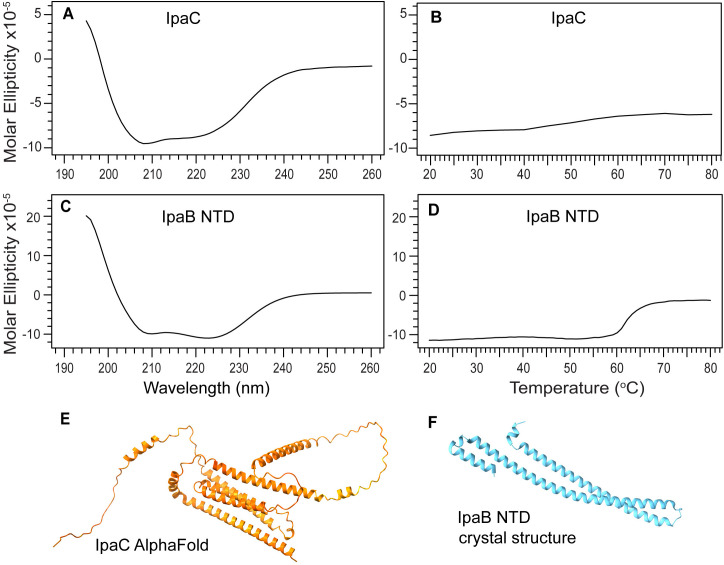
Far-UV CD, thermal denaturation, and structures of IpaC and IpaB NTD. A) Far-UV CD of full-length IpaC shows helical character with minima at 208 and 222 nm that are characteristics of alpha helical proteins. B) Thermal denaturation of full-length IpaC at 222 nm does not show a melting temperature indicative of lack of tertiary structure. C) Far-UV CD of IpaB N-terminal domain. D) Thermal denaturation of IpaB NTD. E) AlphaFold prediction of IpaC following AlphaFold coloring scheme (orange denotes low confidence prediction). F) Crystal structure of IpaB residues 74-242, PDB ID 5wkq.

When urea is exhaustively removed, the CD of purified IpaC shows an alpha helical protein without any random coil conformation (Fig 6A). This CD spectrum (Fig 6A) is similar but not identical to the CD spectrum of IpaC purified under native conditions by Birket et al. [8], which showed a bit of random coil character as indicated by a lower peak at 208 nm compared to the 222 nm peak. We attribute the bit of random character in the CD spectrum of Birket et al. [8] to the longer 20-residue His-tag used by Birket et al. [8], compared to the shorter 8-residue His-tag in our construct. It is thus possible to express and purify IpaC as inclusion bodies and denaturation in 6 M urea for purification, followed by stepwise dialysis and exhaustive centrifugal filtration to remove any traces of urea to obtain a CD spectrum of IpaC that is predominantly alpha helical in character and without random coil. Since urea absorbs strongly in the far-UV, if the CD spectra must be truncated at 200 nm, further removal of urea is required to acquire CD below 200 nm in the far UV region.

The thermal denaturation plot of IpaC as monitored by CD at 222 nm showed no melting temperature (Fig 6B). This behavior is similar to alpha helical proteins that do not fold into three-dimensional structures [26, 27]. To confirm this behavior, we used IpaB N-terminal domain, an alpha-helical protein with a known three-dimensional structure as control. IpaB NTD showed the characteristic alpha-helical CD with a prominent double minima at 208 and 222 nm (Fig 6C). The thermal denaturation of IpaB NTD showed an inflection point with a melting temperature (Tm) of 63 °C. (Fig 6D), in contrast with the thermal denaturation of IpaC (Fig 6B). AlphaFold [28] predicted a highly alpha-helical protein for IpaC (Fig 6E), and the crystal structure of IpaB NTD [29] shows an elongated molecule with packed 3 helices (Fig 6F). Results of the CD thermal denaturation indicate that the alpha helices of IpaC do not interact with each other to form into three-dimensional structure.

**Fig 7:**
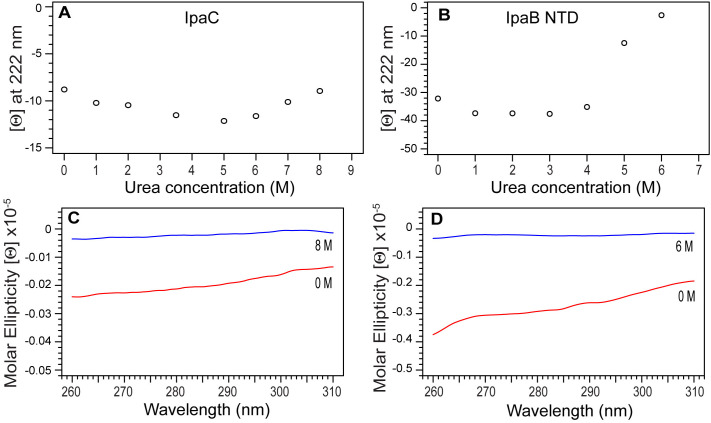
Urea denaturation and near-UV CD of IpaC and IpaB NTD. Change in molar ellipticity [Θ] at 222 nm with increasing urea concentrations for (A) IpaC and (B) IpaB NTD. Near-UV CD scan of (C) IpaC at 0 and 8 M urea and (D) IpaB at 0 and 6 M urea.

**Fig 8:**
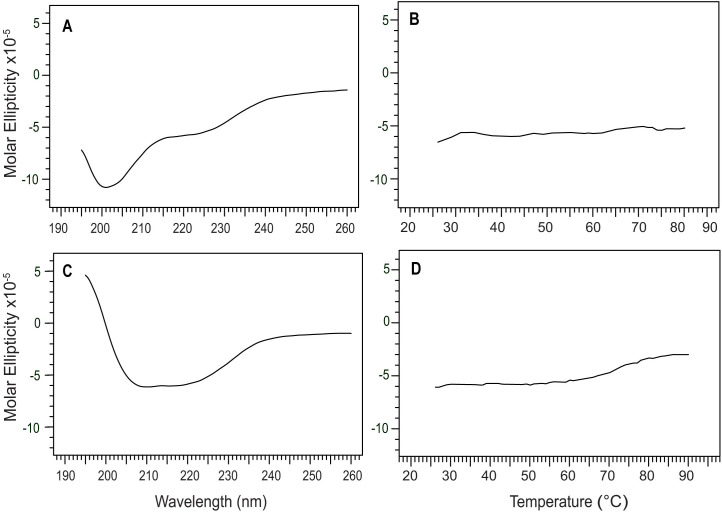
Far-UV CD of IpaC NTD and CTD domains. A) Far-UV CD of the IpaC NTD (residues 1-100) shows predominantly random coils, but some helical character is notable as well. C) Far-UV CD of IpaC CTD (residues 173-363) shows helical character. Thermal denaturation of (B) IpaC NTD and (D) IpaC CTD show lack of melting temperature indicative of lack of tertiary structures for these domains.

We used urea-induced denaturation as monitored by CD spectroscopy at far-UV and near-UV to further characterize IpaC and IpaB (Fig 7). The far-UV CD of IpaC in solutions of up to 8 M urea showed that it still has helical content at 222 nm (Fig 7A), in contrast with IpaB NTD which unfolded completely in 6 M urea, starting around 4 M urea (Fig 7B). The near-UV CD scan at 0 and 6 M urea for IpaC (Fig 7C) or 8 M urea for IpaB NTD (Fig 7D) are shown. Upon adding urea, we observed an initial increase in helical content as indicated by the decrease in the molar ellipticities at 222 nm for IpaC (Fig 7A) and IpaB NTD (Fig 7B), which then subsided with increasing urea. This is likely explained by the proteins becoming molten globules as intermediate unfolding states as seen, for example, with serum albumin [30] and carbonic anhydrase II [31]. The resistance of IpaC to unfolding in urea is likely explained by the presence of its highly hydrophobic transmembrane helix creating internal rigidity as seen in members of the Major Facilitator Superfamily of transmembrane proteins, in particular LacY-C154G, XylE, and PepTso [32].

### CD of IpaC NTD and IpaC CTD

We also expressed and purified smaller fragments of IpaC, the N-terminal domain (NTD), and the C-terminal domain (NTD). We used the putative transmembrane (TM) region as the dividing line to make the IpaC NTD and CTD constructs. Both IpaC NTD and IpaC CTD reported herein do not contain the TM-region and are thus expected to be more soluble. The CD spectrum of IpaC NTD showed a predominantly random coil behavior plus an alpha-helical peak at 222 nm (Fig 8A). The CD thermal denaturation of IpaC NTD did not show a melting temperature (Fig 8B). IpaC NTD is thus predominantly random coil, with a portion that is alpha-helical. On the other hand, the CD of IpaC CTD showed a predominantly alpha-helical domain (Fig 8C). The CD thermal denaturation of IpaC CTD also did not show a melting transition (Fig 8d). Unfortunately, the smaller IpaC constructs containing the TM-region, IpaC residues 1–173 and IpaC residues 101–363, failed to express in *E. coli* suggesting the introduction of hydrophobic TM-region in the IpaC constructs made them cytotoxic to *E. coli*.

### CD of SipC

**Fig 9:**
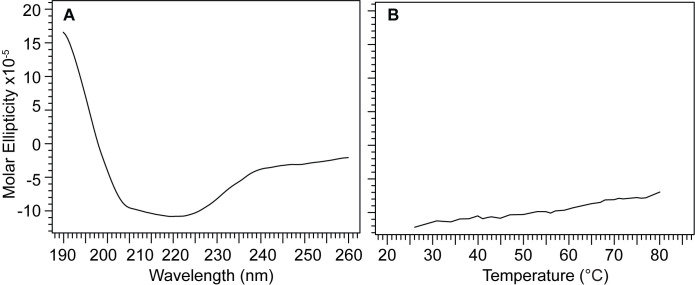
Far-UV CD of SipC. A) Far-UV CD of SipC shows helical character with a minima at 208 nm and 222 nm. B) Thermal denaturation of SipC at 222 nm does not show a melting temperature (Tm), indicating lack of a tertiary structure.

The far-UV CD scan of SipC showed a characteristic alpha-helical protein with peaks at 208 and 222 nm that were almost equal in peak intensity (Fig 9A). The CD scan of SipC did not show traces of random coil behavior. The CD thermal denaturation monitored at 222 nm of SipC did not show a melting temperature and remained essentially a flat line (Fig 9B). These results from CD suggested that SipC is predominantly an alpha-helical protein, but it lacks a three-dimensional structure. The data reported herein are currently the only available CD data in the literature for SipC. Our CD results suggested that the ^13^C/^15^N-based protein NMR methods were not feasible for SipC because of its poor solubility where it could only be kept at 2 uM in solution from a 2 L prep culture. The lack of a three-dimensional structure for SipC also suggested that X-ray crystallography of SipC was not feasible.

### CD of BipC, BipC NTD, and BipC CTD

**Fig 10:**
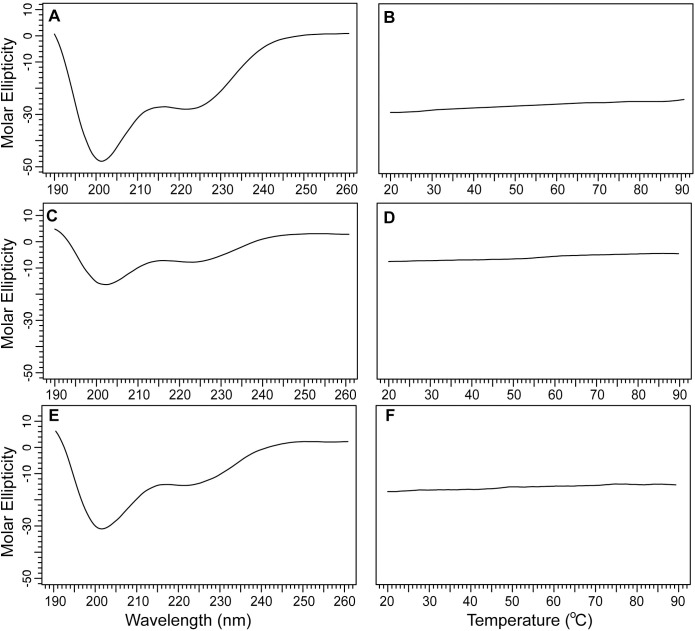
Far-UV CD and thermal denaturation of BipC and BipC domains. Far-UV CD spectra and thermal denaturation of **(A, B)** full-length BipC, (C, D) BipC NTD (residues 1-200) and (E, F) BipC CTD (residues 197-419). CD spectra of (A) BipC, (C) BipC NTD, and (E) BipC CTD show predominantly random coil with alpha helical character. Thermal denaturation at 222 nm for (B) BipC, (D) BipC NTD, and (F) BipC CTD does not show melting temperature indicating lack of tertiary structures.

Full-length BipC and the smaller fragments BipC NTD and BipC CTD were expressed and purified under native conditions and were all soluble proteins. Further, the expression constructs used herein resulted in minimal cloning artifact, with only 2 extra residues in the purified proteins. The CD spectra of BipC indicated a protein that contains both random coil segments and alpha helical secondary structures (Fig 10A). The random coil content in BipC is significant based on the dominant random coil CD peak at 200 nm. CD thermal denaturation did not show a melting temperature for BipC (Fig 10B) indicating that the alpha helices of BipC do not interact with each other to fold into a three-dimensional structure.

We expressed and purified smaller fragments of BipC – BipC NTD and BipC CTD – with the putative TM-region expected at 199–220 by TMPred [33] as the dividing region. BipC NTD did not contain the TM-region, whereas BipC CTD contains the TM-region. Results of CD spectroscopy for BipC NTD (Fig 10c) and BipC CTD (Fig 10E) showed these protein domains behaved similarly to full-length BipC. The CD spectra of BipC NTD (Fig 10C) and BipC CTD (Fig 10E) showed random coil segments and alpha helical contents, and both domains did not show a three-dimensional structure based on the lack of melting temperatures in their CD thermal denaturation plots (Fig 10D and Fig 10F). CD spectroscopy of the smaller fragments of BipC indicated that the alpha helical content of BipC was not due solely on the alpha helix formed by the transmembrane alpha helical region. The BipC NTD construct did not contain the putative TM-region, yet its CD spectrum showed alpha helical content (Fig 10E).

### 2D NMR of IpaC and BipC

**Fig 11:**
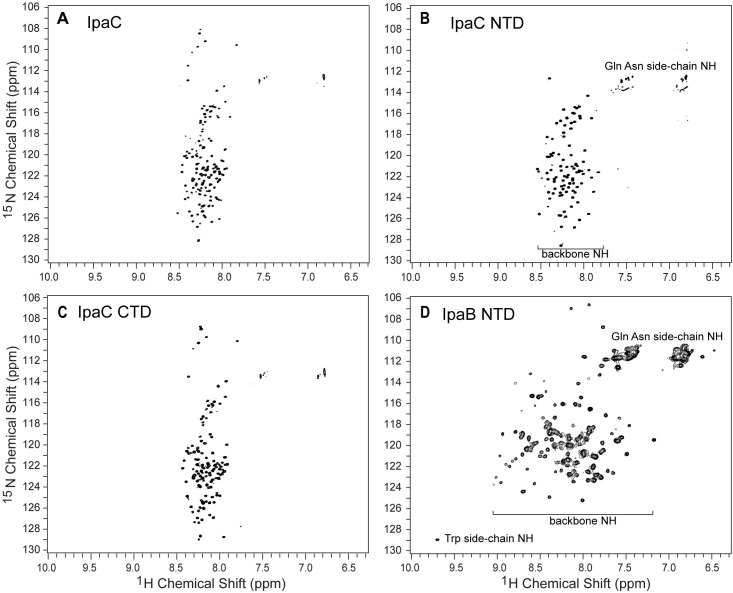
Protein 2D NMR of IpaC and IpaB. NMR ^1^H-^15^N TROSY spectra for IpaC and IpaB. The narrow range of chemical shift of about 1 ppm for the backbone amide (NH) of **(A)** full-length IpaC, (B) IpaC NTD, and (C) IpaC CTD are characteristics of alpha-helical and random coil proteins. In contrast, (D) IpaB NTD, an alpha-helical protein with a three-dimensional structure, shows dispersion of the ^1^H chemical shift to about 2 ppm.

**Fig 12:**
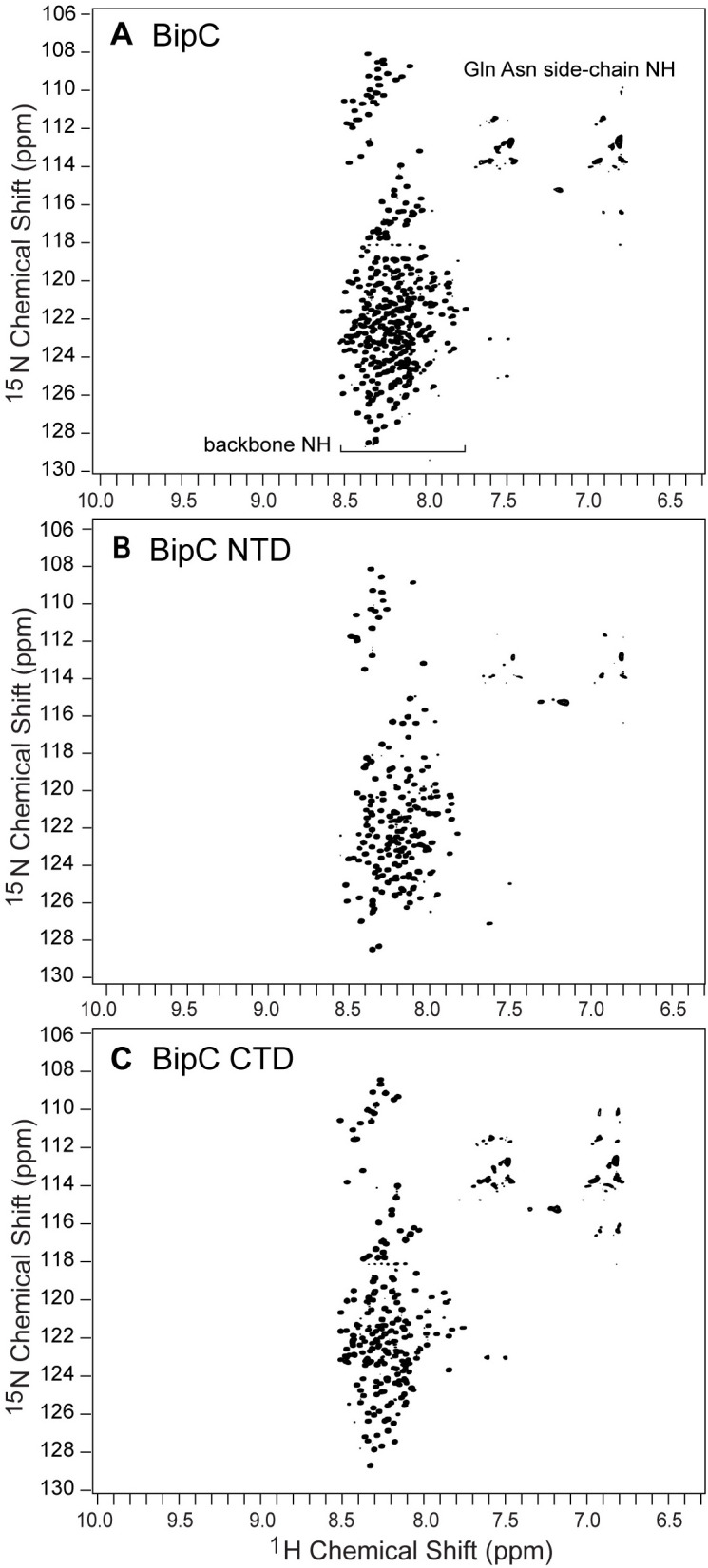
Protein 2D NMR of BipC. 2D ^1^H-^15^N TROSY NMR spectra of (A) BipC, (B) BipC NTD, and (C) BipC CTD. The narrow range of about 1 ppm for the backbone ^1^H proton chemical shifts are characteristics of alpha-helical and random coil proteins.

We were able to express and purify ^15^N-labeled IpaC and BipC at submillimolar concentrations that allowed the acquisition of 2D NMR spectra for these proteins. The poor solubility of SipC precluded the acquisition of 2D NMR data for SipC. The 2D ^1^H-^15^N TROSY of full-length IpaC (Fig 11A), IpaC NTD (Fig 11B), and IpaC CTD (Fig 11C) showed narrow peak dispersions of less than 1 ppm in the proton dimension of the backbone amides. The narrow proton peak dispersion in the 2D ^15^N NMR spectra of proteins is characteristic of alpha-helical proteins and proteins that lack three-dimensional structures [21,26,27]. For comparison, the 2D ^1^H-^15^N TROSY of IpaB NTD (Fig 7D) showed a wider proton chemical shift dispersion of 2 ppm. Some peaks were broad and some were sharp and distinct. The broader dispersion of the proton chemical shift in the 2D ^15^N NMR spectra of IpaB NTD is a characteristic of a protein with a three-dimensional structure. Full-length BipC yielded 2D ^15^N NMR spectra (Fig 12A) with distinct as well as overlapping peaks. The chemical shift dispersion for the proton backbone amides of BipC (Fig 12) was narrow, with less than 1 ppm (between 7.9 ppm to 8.5 ppm), indicating that BipC lacks a three-dimensional structure. Likewise, the 2D ^15^N NMR spectra of BipC NTD (Fig 12B) and BipC CTD (Fig 12C) showed similar characteristics to full-length BipC (Fig 12A), with spectra characterized by narrow proton chemical shift range of less than 1 ppm, indicating that these domains of BipC lacked a three-dimensional structure.

## Discussion

There are currently no three-dimensional structures for any of the T3SS minor translocases. AlphaFold [28] predicts that IpaC (Fig 6E), SipC, and BipC form long alpha helices that arrange into three-dimensional structures forming alpha helical bundles. However, these predicted models have low confidence. Likewise, a template-based homology modeling showed that BipC folds into a tertiary structure with long alpha helical bundles [17]. Thus, prior to our results presented here, the current understanding of the structures of IpaC, SipC and BipC is that they are alpha-helical proteins that form into helical bundles, suggesting they form into three-dimensional structures. The main contribution of our CD (Figs 6-10) and NMR (Figs 11 and 12) data presented here is that IpaC, SipC, and BipC are alpha helical proteins with no tertiary folds. However, these proteins are not intrinsically disordered as IpaC (Fig 6) and SipC (Fig 9) have predominantly alpha helical secondary structures, whereas BipC is a mix of alpha helices and random coil (Fig 10) indicating the presence of intrinsically disordered regions in BipC. Our CD and NMR results showing that IpaC, SipC, and BipC have no well-defined three-dimensional structures explain why these proteins have resisted atomic structural determination by crystallography or cryo-EM, and why AlphaFold yielded low confidence structural models for these proteins.

The minor translocases (IpaC, SipC, BipC) together with the major translocases (IpaB, SipB, BipB) assemble on the host cell membrane to form the translocon [1]. The implication of our results suggests that the translocon is assembled from a partially folded minor translocase. It is currently unknown if the minor translocase undergoes folding transition into a more well-defined three-dimensional structure upon insertion into membrane and assembly into the translocon. Prior results by others suggested possible structural changes in IpaC when placed in a membrane mimetic. IpaC in 0.1% Tween 20 showed a CD melting temperature of 43 °C, suggesting stabilization of the structure of IpaC in micelles [20]. Tween 20 is a non-ionic detergent with a critical micelle concentration of 0.072% that is used in stabilizing membrane proteins [34]. Others showed by CD increased alpha helical content of IpaC in OPOE and LDAO detergent micelles [7], and decreased amount of random coil relative to the alpha helical content of IpaC in 50:50 DOPC/DOPG liposomes [9]. These results suggest that the membrane environment may influence the folding of the minor translocases.

Our results provide new biophysical CD and NMR data that help understand the structures for a major sub-family of the minor translocases. The phylogenetic tree [1] of the minor translocases show a common ancestor with 3 sub-families, formed by the clade of *Burkholderia*, *Shigella*, and *Salmonella; Escherichia,* forming its own clade*;* and a third clade formed by *Yersinia* and *Pseudomonas.* Within the *Burkholderia* clade, *Shigella* and *Salmonella* both share a more recent common ancestor than they do with *Burkholderia*. This trend is born out in our results. BipC is a soluble protein (Fig 5), whereas IpaC (Fig 1) and SipC (Fig 4) go to inclusion bodies. BipC is the most soluble in this clade, followed by IpaC, then SipC as the least soluble. BipC consists of alpha-helices and intrinsically disordered regions (Fig 10), whereas IpaC (Fig 6) and SipC (Fig 9) are predominantly alpha-helical in secondary structure without a significant amount of random coil. Also worth noting is that IpaC and SipC are more similar physically as well. IpaC is approximately 38 kD and SipC is 37 kD, whereas BipC is significantly larger at 45 kD. The expression and purification, CD, and NMR data for the minor translocases of *Escherichia, Yersinia,* and *Pseudomonas* remain unknown.

We used CD spectroscopy to determine the secondary structures of the IpaC-family of proteins (Figs 6-10). When combined with thermal denaturation, CD can inform on whether an alpha helical protein has a tertiary structure based on the melting plot of a CD thermal denaturation scan monitored at 222 nm, as seen in this example of a coiled-coil protein [35]. An alpha helical protein with a tertiary structure shows a sigmoidal-shaped transition with a melting temperature (Tm), as in the case for IpaB NTD (Fig 6). In contrast, alpha helical proteins where helices do not interact with each other do not show a melting transition, as seen in other partially-folded T3SS alpha-helical proteins for the inner rod [21], and the chaperone proteins LcrG [26] and PcrG [27].

What hampered the structural biology of the minor translocases are the challenges in the expression and purification these proteins. IpaC (Figs 1-3) and SipC (Fig 4) form inclusion bodies when expressed in *E. coli*, and required urea-solubilization for purification. IpaC and SipC are also poorly soluble and prone to aggregation in water [11], with IpaC requiring stabilization in 200 mM urea [7,9] or 0.1% Tween 20 [20] for CD spectroscopy. Thus, prior CD data in the literature for IpaC showed an alpha-helical protein with a significant amount of random coil [7,9] because it required solubilization in 200 mM urea. The CD thermal denaturation of IpaC in 200 mM urea did not show a melting temperature [7]. IpaC showed a CD melting temperature of 43 °C in 0.1% Tween 20 micelles [20], so it remained unknown if the melting transition is due to IpaC tertiary structure or to stabilization of IpaC in Tween 20 micelles. When IpaC was co-expressed with its chaperone IpgC, the IpaC-IpgC complex is soluble and can be purified under native conditions allowing the acquisition of the CD spectrum of free IpaC and the CD thermal denaturation of the IpaC-IpgC complex [8]. The CD spectrum of free IpaC by Birket et al. [8] showed a predominantly alpha-helical protein without the random coil CD peak at 205 nm [7,9]. However, the CD thermal denaturation for this native IpaC was not done [8], so it remained unknown if IpaC has a tertiary structure outside of a membrane mimetic. There are currently no CD spectra in the literature for SipC. Osiecki et al. [11] reported the expression of SipC, but it was practically insoluble requiring 200 mM urea to keep it soluble, and the CD spectra for SipC was not reported. Kang et al. [17] reported the CD spectrum and CD thermal denaturation of recombinant BipC. The BipC construct had a 48-residue tag, representing 11% of the length of the protein, thus it was not possible to conclude if the observed random coil behavior of BipC was due to the tag, or inherent in native BipC.

An important consideration in the urea-solubilization of IpaC and SipC inclusion bodies is the complete removal of urea during protein purification. The intense absorbance of urea even at millimolar concentrations [36] precludes the acquisition of far-UV CD spectra below 200 nm. Hence, prior CD spectra reported for IpaC in 200 mM urea to keep it soluble started at 200 nm, and the resulting CD spectra contain random coil CD peak near 205 nm [7,9]. We reasoned that CD spectroscopy can be used as a readout of the exhaustive and complete removal of urea during the urea-purification of IpaC inclusion bodies by acquiring CD spectra below 200 nm. We used several cycles of Amicon filtration to exchange IpaC and SipC into urea-free buffer until the intense urea CD band below 200 nm disappeared. (This took about 6 cycles of Amicon filtration.) This allowed the acquisition of the far-UV CD spectra of IpaC (Fig 6) starting from 195 nm and SipC (Fig 9) from 190 nm, showing that these proteins are predominantly alpha-helical in secondary structures and without the random coil CD peak near 205 nm. The CD spectra of IpaC purified under native conditions by Birket et al. [8] started at 190 nm and showed a predominantly alpha helical secondary structure. Future studies can use CD spectroscopy to monitor the complete removal of urea to obtain recombinant IpaC and SipC that are predominantly alpha-helical in secondary structures.

The advantages of the IpaC and SipC constructs used herein are the short 8-residue His-tag, and the single-step purification by nickel affinity chromatography. The reported co-expression of IpaC with IpgC required a two-step purification by nickel affinity chromatography followed by gel filtration chromatography, and removal of IpaC from the IpaC-IpgC complex by dropping to pH 5 [8]. Additionally, the expression and purification of the subdomains of IpaC (Fig 2, Fig 3) and BipC (Fig 5) reported herein should be useful in using reductionist methods such as NMR to query the structural biology of these minor translocases.

## Conclusion

Currently, the atomic structures are unknown for any member of the family of the T3SS minor translocases that assemble into the translocon of the T3SS. Our CD and 2D NMR data presented above for the sub-family of the minor translocases *Shigella* IpaC, *Salmonella* SipC, and *Burkholderia* BipC, show that these proteins are partially folded alpha-helical proteins lacking in well-defined three-dimensional structures. IpaC and SipC are predominantly alpha-helical in secondary structures, whereas BipC is a mix of alpha-helices and random coil secondary structures. Our results suggest that the translocon is assembled from proteins that are lacking in tertiary structures.
